# Differential expression of genes encoding proteins of the HGF/MET system in insulinomas

**DOI:** 10.1186/s13098-015-0079-3

**Published:** 2015-10-01

**Authors:** Cahuê De Bernardis Murat, Paula Waki Lopes da Rosa, Maria Angela Henriques Zanella Fortes, Luciana Corrêa, Marcel Cerqueira Cesar Machado, Estela Maria Novak, Sheila Aparecida Coelho Siqueira, Maria Adelaide Albergaria Pereira, Maria Lucia Corrêa-Giannella, Daniel Giannella-Neto, Ricardo Rodrigues Giorgi

**Affiliations:** Laboratório de Endocrinologia Celular e Molecular (LIM-25) do Hospital das Clínicas da Faculdade de Medicina da Universidade de São Paulo (HC-FMUSP), Av. Dr. Arnaldo, 455, 01246-903 São Paulo, SP Brazil; Departamento de Patologia Oral, Faculdade de Odontologia da Universidade de São Paulo, São Paulo, Brazil; Laboratório de Emergências Clínicas (LIM-51) da FMUSP, São Paulo, Brazil; Laboratório de Biologia Molecular da Fundação Pró-Sangue Hemocentro de São Paulo, São Paulo, Brazil; Divisão de Patologia do HC-FMUSP, São Paulo, Brazil; Divisão de Endocrinologia do HC-FMUSP, São Paulo, Brazil; Centro de Terapia Celular e Molecular (NUCEL/NETCEM) da FMUSP, São Paulo, Brazil; Programa de Pós-Graduação em Medicina, Universidade Nove de Julho—UNINOVE, São Paulo, Brazil; Programa de Pós Graduação em Ciências da Saúde, Universidade de Santo Amaro (UNISA), São Paulo, Brazil

**Keywords:** Insulinoma, Hepatocyte growth factor, MET receptor, Gene expression, Somatic mutation

## Abstract

**Background:**

Insulinomas are the most common functional pancreatic neuroendocrine tumors, whereas histopathological features do not predict their biological behaviour. In an attempt to better understand the molecular processes involved in the tumorigenesis of islet beta cells, the present study evaluated the expression of genes belonging to the hepatocyte growth factor and its receptor (HGF/MET) system, namely, *MET*, *HGF*; *HGFAC* and *ST14* (encode HGF activator and matriptase, respectively, two serine proteases that catalyze conversion of pro-HGF to active HGF); and *SPINT1* and *SPINT2* (encode serine peptidase inhibitors Kunitz type 1 and type 2, respectively, two inhibitors of HGF activator and of matriptase).

**Methods:**

Quantitative real-time reverse transcriptase polymerase chain reaction was employed to assess RNA expression of the target genes in 24 sporadic insulinomas: 15 grade 1 (G1), six grade 2 (G2) and three hepatic metastases. Somatic mutations of *MET* gene were searched by direct sequencing of exons 2, 10, 14, 16, 17 and 19.

**Results:**

Overexpression of *MET* was observed in the three hepatic metastases concomitantly with upregulation of the genes encoding HGF and matriptase and downregulation of *SPINT1*. A positive correlation was observed between *MET* RNA expression and Ki-67 proliferation index while a negative correlation was detected between *SPINT1* expression and the mitotic index. No somatic mutations were found in *MET* gene.

**Conclusion:**

The final effect of the increased expression of HGF, its activator (matriptase) and its specific receptor (MET) together with a decreased expression of one potent inhibitor of matriptase (SPINT1) is probably a contribution to tumoral progression and metastatization in insulinomas.

**Electronic supplementary material:**

The online version of this article (doi:10.1186/s13098-015-0079-3) contains supplementary material, which is available to authorized users.

## Background

Insulinomas are the most common functional pancreatic neuroendocrine tumors (pNETs) with an estimated incidence of 0.4 per 100,000 person-years [1]. They are usually benign, small, solitary and sporadic and are classified as malignant in the presence of local invasion or distant metastases, whereas histopathological features do not predict their biological behaviour [2].

Several studies have reported molecular alterations in the system comprising hepatocyte growth factor (HGF) and its tyrosine kinase receptor MET (HGF/MET system) in different types of neoplasias [3, 4], in which inappropriate MET activation enhances proliferation, anti-apoptotic events, invasiveness and metastatization [5, 6]. The HGF/MET system has not been systematically investigated in insulinomas.

In an attempt to better understand the molecular processes involved in the tumorigenesis of islet beta cells, the aim of the present study was to assess the mRNA expression of genes belonging to the HGF/MET system in sporadic insulinomas and to correlate the expression findings with histopathological characteristics of the tumors. Besides *MET* and *HGF*, we also evaluated mRNA expression of the following components: (1) *HGFAC* and *ST14*, that encode HGF activator and matriptase, respectively, two serine proteases that catalyze conversion of pro-HGF to active HGF and (2) *SPINT1* and *SPINT2*, that encode serine peptidase inhibitors Kunitz type 1 and type 2, respectively, two potent inhibitors of HGF activator and of matriptase. To evaluate possible mechanisms involved in MET overexpression, the presence of somatic mutations in this gene was also examined.

## Methods

### Patients and tissue specimens

Tissue collection was performed in compliance with the Institutional Ethics Committee (CAPPesq) and in accordance to the Declaration of Helsinki, with informed consent being required from each subject. From 1999 to 2011, 24 tumor tissues were obtained and processed as previously described [7]. Tumor fragments were collected in sterile containers and immediately frozen in liquid nitrogen. The tumors were graded according to the classification system recommended by the European Neuroendocrine Tumor Society (ENETS) and the World Health Organization (WHO): (1) well-differentiated grade 1 neuroendocrine tumor (G1; <2 mitoses/10 HPF [high-power fields] and <3 % Ki-67 index); (2) well-differentiated grade 2 neuroendocrine tumor (G2; 2–20 mitoses/10HPF or 3-20 % Ki-67 index) and; (3) poor-differentiated grade 3 neuroendocrine carcinoma (G3; >20 mitoses/10HPF or >20 % Ki-67 index) [8]. The histopathological characteristics of these insulinomas are shown in Additional file 1: Table S1. The present series consisted of 15 G1 insulinomas, six G2 insulinomas and three hepatic metastases.

### Quantitative real-time reverse transcriptase polymerase chain reaction (qRT-PCR)

Total RNA was extracted using the TriZol reagent (Invitrogen, Carlsbad, CA, USA) according to the manufacturer’s recommendations. RNA integrity and quantity were evaluated using the RNA 6000 Nano Assay with the Agilent 2100 Bioanalyzer (Agilent Technologies, Palo Alto, CA, USA) and only samples with an RNA Integrity Number (RIN) >7 were used. Complementary DNA (cDNA) was synthesized from total RNA. Briefly, first-strand cDNA synthesis was carried out with 1 μg of RNA, 1 μL of oligo(dT) primers (0.5 μg/μL), 1 μL of a solution with all four deoxyribonucleoside triphosphates (each at 10 mM), and 10 × Superscript III reverse transcriptase (Invitrogen).

For TaqMan-based qRT-PCR, 100 ng of cDNA was added to 10 μL of 2 × Universal PCR Master Mix and to 1 μL of 20 × the specific primers and probe set (Applied Biosystems, Carlsbad, CA, USA). Fifty cycles of amplification were performed at 95 °C (15 s) and 60 °C (1 min) in a StepOne Plus Realtime PCR system (Applied Biosystem). The following Assay on Demand primers and probes were used: Hs00300159_m1 (*HGF*), Hs01565584_m1 (*MET*), Hs00173526_m1 (*HGFAC*), Hs01058386_m1 (*ST14*), Hs00173678_m1 (*SPINT1*), Hs01070442_m1 (*SPINT2*) and Hs01652481_g1 (*PSMC6*, which encodes the proteasome 26S subunit, ATPase, 6) used as a control endogenous gene, as previously validated [7]. Human liver cDNA was used as positive control for *HGF*, *MET*, *HGFAC* and *ST14* genes expression and human placental tissue was used as a positive control for *SPINT1* and *SPINT2* genes expression. All samples were run in triplicate. Gene expression levels were analyzed by the mathematical model variation described by Livak and Schmittgen [9], 2^−ΔCt^ [10].

### Mutational analysis

DNA from tumoral samples was extracted using the DNeasy kit (Qiagen, Valencia, CA, USA) according to the manufacturer’s instructions. For mutational analyses, exons 2, 10, 14, 16, 17 and 19 of *MET* proto-oncogene (GenBank accession number NM_000245) were amplified with specific primers (designed using the Primer 3 software) for direct sequencing on an ABI 3130X Genetic Analyzer (Applied Biosystems) in 50 μl of PCR reaction mixture [100 ng genomic DNA, 0.2 mM of each primer, 200 μM deoxynucleotides, 1× buffer and 1 U DNA Taq polymerase (GE Healthcare, Salt Lake City, UT, USA)]. PCR products were purified with ExoSAP IT (USB, Cleveland, OH, USA) and sequenced using the BigDye Terminator v1.1 Cycle Sequencing Kit (Applied Biosystems). Cycling conditions were as follows: 95 °C for 5 min, 35 cycles of 95 °C for 30 s, followed by 56 °C (all exons) for 30 s and 72 °C for 10 min in a Thermocycler Model Veriti (Applied Biosystems). The sequences were analyzed using the Sequencher software, version 4.10.1.

### Statistical analysis

Statistical tests were performed with JMP Version 10 statistical computer program (SAS Institute, Cary, NC, USA). Data were evaluated by Kruskal–Wallis test followed by Dunn’s multiple comparisons test. Analyses of correlations between the values of each gene expression and histopathological features were performed with the Spearman correlation test. Statistical significance was fixed at probability levels of <0.05.

## Results

### Gene expression by qRT-PCR

**Fig. 1:**
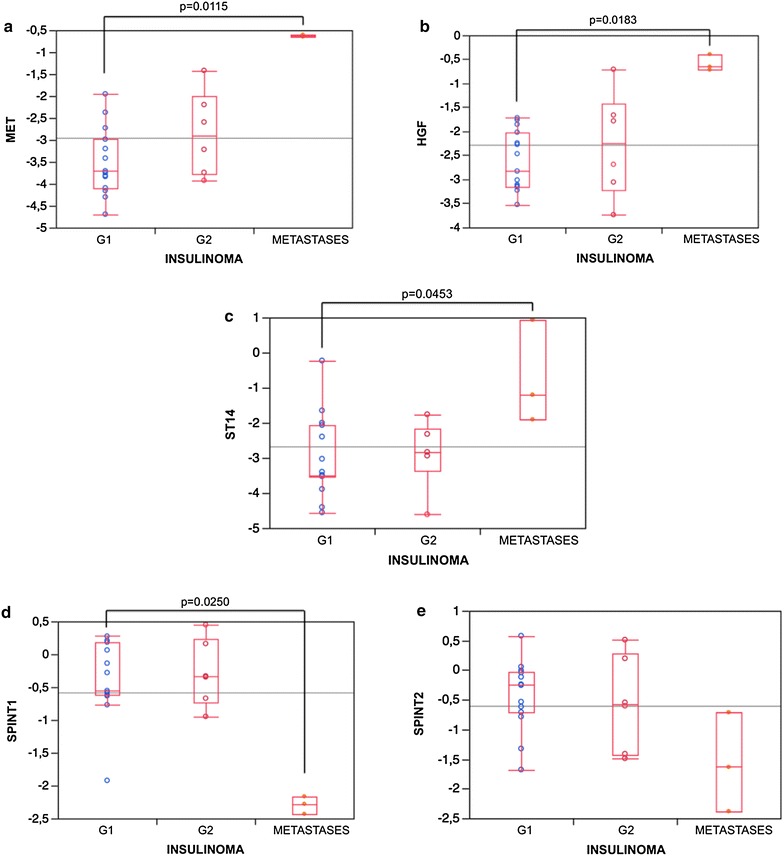
mRNA expression values of *MET* (**a**), *HGF* (**b**), *ST14* (**c**), *SPINT1* (**d**), and *SPINT2* (**e**) in insulinomas graded according to 2014 ENETS/WHO classification (G1, G2 and hepatic metastases). *Box diagram* comparing relative mRNA expression levels of genes; the *horizontal line* within the *box plot* represents the median value, the *box plot* limits refer to 25th–75th percentiles, and the *box plot bars* include the 10th–90th percentiles for mRNA levels

As shown in Fig. 1, a higher expression of *MET* (*P* = 0.0115, Panel A), *HGF* (*P* = 0.0183, Panel B) and *ST14* (*P* = 0.0453, Panel C) mRNA was observed in the three metastases in comparison to G1 insulinomas. No *HGFAC* gene expression was detected in any studied tumoral sample (data not shown). The expression of *SPINT1* mRNA (Panel D) was lower in the three metastases in comparison to G1 insulinomas (*P* = 0.0250), whereas no difference was detected in *SPINT2* gene expression (Panel E). No statistically significant differences were observed between G1 and G2 insulinomas for any of the studied genes.

Additional file 2: Table S2 depicts the statistically significant correlations observed between tumoral histopathological variables and mRNA expression of the studied genes and also the correlations found among mRNA expression of the target genes. A positive correlation was observed between *MET* mRNA expression and Ki-67 proliferation index (ρ = 0.4682; *P* = 0.0210) while a negative correlation was detected between *SPINT1* mRNA expression and the mitotic index (ρ = −0.4721; *P* = 0.0174). High positive correlations were found between *MET* and *HGF* mRNA expression (ρ = 0.7827; *P* < 0.0001) and between *MET* and *ST14* mRNA expression (ρ = 0.8272; *P* < 0.0001).

### MET mutation analysis

Direct sequencing of the exons previously found mutated in other neoplasias (2, 10, 14, 16, 17 and 19) did not reveal somatic mutations in the *MET* gene in any tumoral sample.

## Discussion

This study demonstrated the differential expression of four genes belonging to the HGF/MET system between G1 insulinomas and liver metastases of insulinomas, suggesting the participation of this pathway in the later stages of tumorigenesis.

The final effect of the increased expression of a growth factor (HGF), one of its activator (ST14) from the precursor molecule and its specific receptor (MET) together with a decreased expression of one potent inhibitor of the growth factor activator (SPINT1) is probably a contribution to tumoral progression, which is consistent with findings in other human neoplasias. In pancreatic carcinoma cells, HGF seems to have a potent role in invasion and metastasis by exerting its antianoikis effect through phosphatidylinositol 3-kinase pathway [11]. In gastric cancers, for instance, the high expression of *MET* and *HGF* is associated with the development of metastases [12].

Findings that corroborate this hypothesis are the positive correlations observed between *MET* RNA expression and the Ki-67 proliferative index as well as the negative correlation between *SPINT1* expression and the mitotic index. The correlations found among the RNA expression of the studied genes suggest interdependence among the different proteins that comprise the HGF/MET system.

In contrast to our findings, Wulbrand et al. [13] did not detect the expression of MET in ten insulinomas. However, only one case was described as metastatic and the methodology employed (RT-PCR) is less sensitive that the qRT-PCR used in the present study. Hansel et al. [14] have previously described increased expression of MET in metastatic *versus* non-metastatic pNETs, as well as in lymph node and in liver metastases, but their series did not include any metastatic insulinoma, only non-functioning tumors, glucagonomas and gastrinomas. These data suggest that overexpression of MET is a molecular marker of malignancy not only of insulinomas, but also of other pNETs.

Activating mutations in the *MET* gene can promote the hyperactivity of this signalling pathway [15]. The following main oncogenic mutations have been described for this gene [4]: point mutations that lead to an alternative splicing, which results in a small protein without a PKC (protein kinase C) binding degradation site [16]; point mutations in the tyrosine kinase domain, which maintain the receptor constitutively active [17], and mutations that inactivate the negative regulatory Cbl (E3 ubiquitin protein ligase) binding site, which drive MET to ubiquitination, endocytosis, and subsequent degradation [18].

To investigate whether the presence of somatic mutations would be involved in *MET* upregulation, direct sequencing of exons previously reported as mutated in other cancer types was performed and no mutations were detected. Alternative explanations for the observed MET overexpression could be gene amplification, as reported in non-small-cell lung carcinoma [19] and in ovarian adenocarcinoma [20], gains of 7q (MET gene is located on 7q31) that are known to be involved in the progression of insulinomas and other pNETs [21], and epigenetic modifications, such as loss of DNA methylation, already identified in a mouse model of hepatocellular carcinoma [22].

An interesting finding of the present study was the absence of RNA expression of *HGFAC,* considered the most powerful activator of pro-HGF in HGF [23], indicating that in insulinomas, matriptase (and maybe other proteins not evaluated in this study, such as hepsin [24] ) is responsible for pro-HGF proteolysis. A lower RNA expression of the inhibitor of matriptase *SPINT1* was detected in the insulinoma metastases, potentially contributing to increased activity of matriptase to participate in HGF activation.

The main limitation of this study is the small number of metastasis samples, due to the rarity of this condition. Nevertheless, the differential expression of not only one but four different genes of HGF/MET system, the observed correlations between *MET* and *SPINT1* mRNA expression and tumor histopathological features and the previous identification of MET as overexpressed in other metastatic pNETs [14] suggest the participation of the HGF/MET pathway in the later stages of insulinoma tumorigenesis. The confirmation of our findings in larger series could allow the development of strategies oriented to this system as a potential target to control the progression of insulinomas.

## Additional files

10.1186/s13098-015-0079-3 Summary of patient's demographic data and tumor histopathological features of the 24 insulinomas.
10.1186/s13098-015-0079-3 Statistically significant correlations among RNA expression of the studied genes and tumoral histopathological variables.
