# Supplementary material for: Differential expression of genes encoding proteins of the HGF/MET system in insulinomas
Source: Diabetol Metab Syndr. 2015 Oct 1;7:84. doi: 10.1186/s13098-015-0079-3 (PMC4591639; doi:10.1186/s13098-015-0079-3)
Supplement: Supplementary file 1 — 10.1186/s13098-015-0079-3 Summary of patient's demographic data and tumor histopathological features of the 24 insulinomas. [file 13098_2015_79_MOESM1_ESM.doc]

Table 1. Summary of patient's demographic data and tumor histopathological features of the 24 insulinomas.

| **#** | **SEX** | **AGE**  **(years)** | **Ki-67 %** | **MITOSIS**  **(/10HPF)** | **TUMORAL SIZE**  **(cm)** | **GRADE / STAGE** |
| --- | --- | --- | --- | --- | --- | --- |
| 1 | M | 42 | 1 | 1 | 12.0 | liver metastasis |
| 2 | M | 60 | 1 | 0 | 1.0 | G1 |
| 3 | F | 42 | 1 | 1 | 0.8 | G1 |
| 4 | M | 51 | 1 | 2 | 1.5 | G2 |
| 5 | M | 73 | 5 | 0 | 2.0 | G2 |
| 6 | M | 25 | 5 | 0 | 2.0 | G2 |
| 7 | M | NA | 2 | 1 | 2.5 | liver metastasis |
| 8 | M | 19 | 1 | 1 | 2.0 | G1 |
| 9 | M | 28 | 1 | 1 | 1.5 | G1 |
| 10 | F | 42 | 1 | 0 | 2.0 | G1 |
| 11 | F | 71 | 1 | 1 | 0.8 | G1 |
| 12 | M | 40 | 2 | 1 | 2.4 | G1 |
| 13 | F | 57 | 5 | 1 | 2.6 | G2 |
| 14 | F | NA | 1 | 1 | 1.0 | G1 |
| 15 | M | 54 | 1 | 0 | 1.7 | G1 |
| 16 | F | 28 | 5 | 4 | 0.7 | G2 |
| 17 | F | 35 | 1 | 0 | 0.8 | G1 |
| 18 | F | 37 | 2 | 0 | 1.2 | G1 |
| 19 | F | 58 | 5 | 1 | 2.6 | G2 |
| 20 | F | 44 | 1 | 0 | 1.4 | G1 |
| 21 | F | 42 | 1 | 0 | 0.9 | G1 |
| 22 | M | 73 | 1 | 0 | 1.8 | G1 |
| 23 | F | 44 | 1 | 0 | 1.5 | G1 |
| 24 | M | 43 | 5 | 3 | 1.0 | liver metastasis |

Legend: F, female; G1, grade 1; G2, grade 2; HPF, high-power fields; M, male; NA, not available.
