# Supplementary material for: Differential expression of genes encoding proteins of the HGF/MET system in insulinomas
Source: Diabetol Metab Syndr. 2015 Oct 1;7:84. doi: 10.1186/s13098-015-0079-3 (PMC4591639; doi:10.1186/s13098-015-0079-3)
Supplement: Supplementary file 2 — 10.1186/s13098-015-0079-3 Statistically significant correlations among RNA expression of the studied genes and tumoral histopathological variables. [file 13098_2015_79_MOESM2_ESM.docx]

Table 2. Statistically significant correlations among RNA expression of the studied genes and tumoral histopathological variables.

| **Variables** | **Rho SPEARMAN**  **(ρ)** | ***P*** |
| --- | --- | --- |
| *MET* X Ki-67 | 0.4682 | 0.0210 |
| *SPINT1* X MITOSIS (/10 HPF) | -0.4721 | 0.0174 |
| *MET* X *HGF* | 0.7827 | <0.0001 |
| *MET* X *ST14* | 0.8272 | <0.0001 |
| *MET* X *SPINT1* | -0.4493 | 0.0115 |
| *SPINT1* X *HGF* | -0.3962 | 0.0358 |
| *SPINT1* X *ST14* | -0.4915 | 0.0116 |
| *ST14*  X *HGF* | 0.5311 | 0.0167 |

Legend: HPF, high-power fields.
